# Immune Clustering Reveals Molecularly Distinct Subtypes of Lung Adenocarcinoma

**DOI:** 10.3390/biomedicines13040849

**Published:** 2025-04-02

**Authors:** Yan Lender, Ofer Givton, Ruth Bornshten, Meitar Azar, Roy Moscona, Yosef Yarden, Eitan Rubin

**Affiliations:** 1 Shraga Segal Department of Microbiology, Immunology & Genetics, Ben-Gurion University in the Negev, Beer Sheba 8410501, Israel; lyan125@gmail.com (Y.L.); givtono@post.bgu.ac.il (O.G.); rutbo@post.bgu.ac.il (R.B.); roymosc@post.bgu.ac.il (R.M.); 2 The Faculty of Medical & Health Sciences, Tel Aviv University, Tel Aviv 6997801, Israel; 3 David Fikes 5, Rehovot 7632805, Israel; meitarazar@gmail.com; 4 Department of Immunology and Regenerative Biology, Weizmann Institute of Science, Rehovot 7610001, Israel; yosef.yarden@weizmann.ac.il

**Keywords:** immunotherapy, refractive tumors, clustering, subtypes

## Abstract

**Background/objectives:** Lung adenocarcinoma, the most prevalent type of non-small cell lung cancer, consists of two driver mutations in KRAS or EGFR. These mutations are generally mutually exclusive and biologically and clinically different. In this study, we aimed to test if lung adenocarcinoma tumors could be separated by their immune profiles using an unsupervised machine learning method. The underlying assumption was that differences in the immune response to tumors are characteristic of tumor subtypes. **Methods:** RNA-seq data were projected into inferred immune profiles. Unsupervised learning was used to divide the lung adenocarcinoma population based on their projected immune profiles. **Results:** The patient population was divided into three subgroups, one of which appeared to contain mostly EGFR patients. The tumors in the different clusters significantly differed in their expression of some of their known immune checkpoints (TIGIT, PD-1/PD-L1, and CTLA4). **Discussion:** We argue that EGFR mutations in each subgroup are immunologically different, which implies a distinct tumor microenvironment and might relate to the relatively high resistance of EGFR-positive tumors to immune checkpoint inhibitors. However, we cannot make the same claim about KRAS mutations.

## 1. Introduction

Lung cancer is the most common cause of death from cancer worldwide, with 1.79 million deaths in 2020 [1]. Non-small cell lung cancer (NSCLC) accounts for 85% of all lung cancers, with 40% of these cases being lung adenocarcinoma (LUAD), 25–30% squamous cell carcinoma, and 5–10% large cell carcinoma [2]. The five-year survival rate of LUAD patients is about 15%, lower than the overall NSCLC five-year survival rate (18%), as it is usually diagnosed in metastatic form [3].

The most common driver mutations in LUAD are Kirsten rat sarcoma virus (KRAS) and Epidermal growth factor receptor (EGFR; ~30% and ~15%, respectively, in western populations) [4,5]. The most common mutation occurs in KRAS, a member of the rat sarcoma (RAS) family. It most commonly involves a gain-of-function mutation (usually resulting from a single base substitution) that activates KRAS, promoting cancer invasion and metastasis. Patients harboring a KRAS mutation usually respond poorly to chemotherapy but are more likely to respond well to immunotherapy [6,7] than patients with the EGFR mutation. It was shown in mice that KRAS mutations are related to increased CD8+ T cell, regulatory T cell, and myeloid cell migration into the tumor [7]. In addition, KRAS-mutated patients carry, on average, a higher tumor mutation burden than EGFR-mutated patients [8].

EGFR is the second most common driver mutation in lung adenocarcinoma (LUAD). This mutation encodes a transmembrane glycoprotein that plays a major role in the cell decision between proliferation and apoptosis [9,10]. The two most common mutations found in the EGFR gene in LUAD tumors include exon 19 in-frame deletions and leucine-to-arginine substitution in exon 21 [10,11,12]. Together, these two mutations account for 85–90% of all EGFR mutations [11,12,13,14,15] and are associated with a low mutation burden [12].

From a clinical perspective, EGFR mutations are usually common in non-smoking females of Asian ethnicity [13], while KRAS mutations are more common in smokers [14], but no differences were detected in the prognosis and diagnosis of patients with tumors harboring either mutation [15]

Since both mutations are clinically too similar upon diagnosis, we sought to find a way to distinguish between them by examining the immune repertoire of patients carrying these mutations. We constructed a pipeline by using unsupervised learning based on “xCell” (see Methods below) [16]. We found that LUAD patients from the Cancer Genome Atlas (TCGA) could be divided into three distinct groups. To test if the groups were molecularly distinct, we checked if driver mutations were randomly distributed across the groups. Most EGFR-mutated patients fell into one cluster, suggesting a possible way to characterize LUAD groups using unsupervised learning.

## 2. Materials and Methods

### 2.1. Study Population

This study was conducted on the Cancer Genome Atlas population, which is described elsewhere [17]. A brief description of the study population is provided in Table 1 below. Mutation data were available for 566 patients and clinical data for 498 patients.

### 2.2. Data

The LUAD mutation data were downloaded from cBioPortal [18,19] on 2 February 2021. Before analysis, outliers were removed based on their mutation count following z-score normalization (z > 3) [20]. The z-score was calculated for EGFR- and KRAS-mutated LUAD patients using single nucleotide variation (SNV) data [21] for total mutation calculation, downloaded on 20 July 2020. Seven samples, ‘05-4382’, ‘17-Z022’, ‘55-7907’, ‘55-8506’, ‘55-A490’, ‘78-7155’, and ‘86-A4JF’, were removed for having exceptionally high z-scores. All subsequent analyses were performed after this removal.

For k-means clustering, the number of clusters was optimized using the knee method [22]. The random seed was set to 42 during the calculations.

For clinical information, data were downloaded on 23 May 2021 from cBioPortal [18,19]. In addition, the EGFR patients’ smoking information was downloaded from Xena GDC [23] (27 May 2021).

Overall survival information was downloaded from cBioPortal [23] on 23 May 2021.

### 2.3. Statistics

To evaluate whether there were statistically significant differences in the clinical characteristics among the identified patient clusters, a chi-square contingency test (Fisher’s exact test) was used to test for the independence of each property in the EGFR wild-type (WT) groups. Testing was performed using the Python programming language (ver. 3.8) with the SciPy library (version 1.7.3). For differential expression analysis, the R programming language (version 4.2.0 [24]) was used with the Deseq2 package (version 1.36.0 [25])

### 2.4. Immune Inference from Bulk Expression Profiles

**Figure 1:**
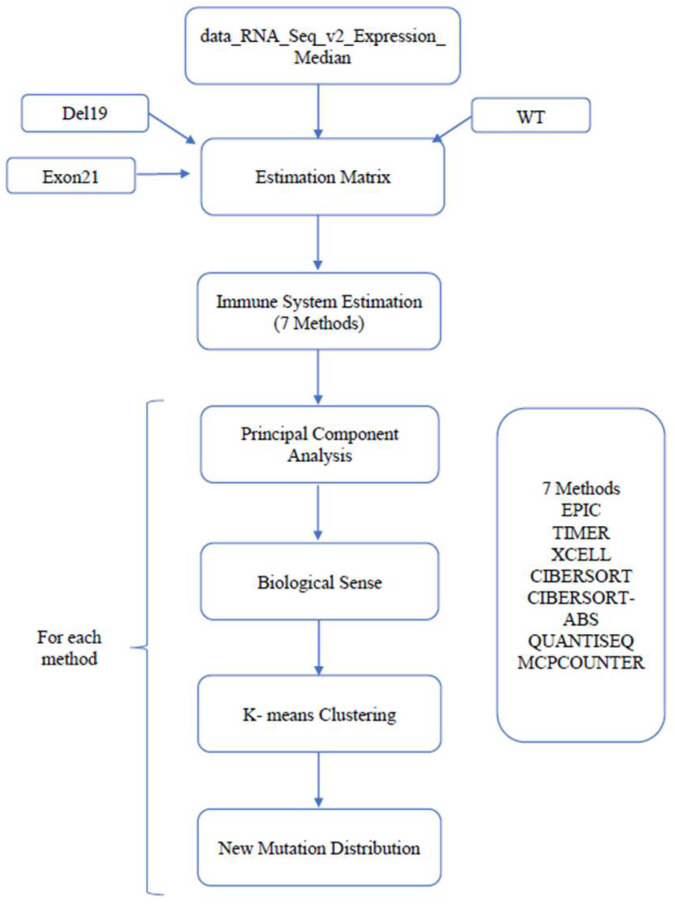
From the expression profiles to a new mutation distribution (based on previous knowledge). This figure describes the pipeline utilized in this work to examine the value of immune inference to find new ways to divide LUAD patients. The new mutation distribution was calculated for each cluster separately.

The measurement of immune cells that infiltrate tumors has the potential to reveal the complex role of the immune system in human cancers, as well as its participation in tumor escape mechanisms and treatment response. Bioinformatics techniques were used to infer immune profiles from RNA-seq expression data. For convenience, we used TIMER (ver. 2.0) [26,27,28,29,30,31,32], a website that uses multiple inference methods to utilize cell-type-specific gene signatures. The default parameters were used (input data as downloaded in bulk on 13 April 2022, LUAD extracted). The normalized RNA-seq profile (see Methods above) was converted to immune-related cell type content using xCell. Four EGFR patients lacked expression data and were removed. The data were scaled using z-score transformation and clustered using the k-means algorithm. Both steps were performed with the scikit-learn library (version 1.1.1) [33] in Python. Only clusters with five or more individuals were maintained. K-means clustering was used to divide the patients, and the resulting clusters were compared (Figure 1). The same pre-processing and clustering procedure was performed on the raw expression data of LUAD (downloaded from FirebrowseR [34] on 13 April 2022) without immune conversion.

xCell is described in detail elsewhere [16]. Briefly, this computational method estimates the cell type abundance of immune and stromal cell types from transcriptomic data. The process is based on an algorithm that compares the expression levels of signature genes that are specific to each cell type with the expression levels of all other genes in the dataset.

### 2.5. Overall Survival

A Kaplan–Meier curve was drawn using the Matplotlib package [35] in Python. Out of 559 patients, 61 were omitted for missing overall survival data (n = 498). A pairwise log-rank test was applied to find differences between the populations.

### 2.6. Differential Expression Analysis

The expression of EGFR-mutated patients was compared between patients assigned to sub0 (n = 33), sub1 (n = 4), and sub2 (n = 25) using DeSEQ2 (version 1.34.0).

### 2.7. Clustering

To identify subgroups of tumors with similar expressions, we used the k-means algorithm with xCell-inferred cell content profiles. For a description of how xCell was used to infer the cell profiles, see Section 2.4 above. K-means was carried out (as implemented in the scikit-learn package version 1.1.1) [33] on the first two components of PCA. PCA and k-means performed with the scikit learn implementation (see above). The number of clusters (*K*) was chosen with the Kneed [22] package in python (version 0.7.0), which implemented a knee analysis (an automated elbow analysis algorithm). This analysis resulted in four clusters (sub0, sub1, sub2, and sub3), but as sub3 contained fewer than 5 samples, it was discarded.

## 3. Results

### 3.1. Characteristics of the Study Population

**Figure 2:**
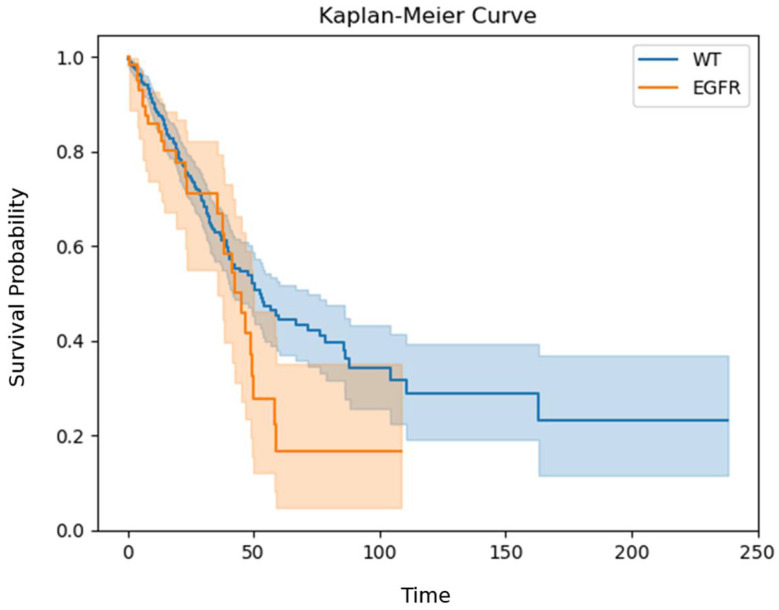
Kaplan–Meier curve of EGFR-mutated and EGFR-WT patients. The Y-axis shows the survival probability, while the X-axis represents the respective time interval. Separate lines, in different colors, each with a band of the same color, represent the comparison of the EGFR-WT (n = 437) vs. EGFR-mutated patients (n = 61); *p* = 0.1; log-rank test.

This study was based on the LUAD subsection of TCGA. The population used in this dataset is described in detail elsewhere [27] (see also Table 1 for a description of the population characteristics). Briefly, it included 558 patients (after integration with mutation data), most of whom were diagnosed at the age of >60 (n = 289). Dividing this population based on EGFR status revealed little gender difference in the EGFR-WT subpopulation (n = 230 vs. 215). In contrast, females were significantly over-represented in the EGFR-mutated patients compared to the EGFR-WT patients (n = 43 vs. 19; *p* = 0.02, chi-square test, Table 1), as previously reported [28]. In terms of ethnicity, this study focused on Caucasians (n = 336), although ethnicity was unknown for ~25% of the population. The diagnosis was mostly at first grade (n = 276), with a strong significant negative correlation between the stage and frequency of detection (R^2^ = 0.9; *p* = 0; Pearson correlation). There were significantly more non-smokers in the EGFR-mutated group than in the WT group (n = 27/66, 46/492, respectively; *p*-value < 0.05; chi-square test), following previous reports (Table 1) [28]. Overall survival was not significantly different in the WT group compared to the EGFR-mutated group (Figure 2; *p* = 0.1; log-rank pairwise test).

### 3.2. Immune Inference and Unsupervised Learning Detect New Groups

To infer the immune status in the tumor from bulk mRNA profiling, we used the xCell tool. Multiple methods were considered to perform this task, and the choice of xCell was mostly arbitrary.

First, we tested the hypothesis that using inferred immune status would reveal groups missed when the raw expression profiles are used. For this, we used the k-means clustering algorithm [29] with the original expression profiles (based on RNA-seq) and the knee analysis method to decide on the number of groups [22]. The population was divided into four subgroups: sub0, sub1, sub2, and sub3. The KRAS-mutated patients were non-randomly distributed among these groups (*p* = 0.01, chi-square test), with sub1 enriched with KRAS-WT patients. On the other hand, no enrichment in EGFR-mutated patients could be detected (*p* = 0.18, chi-square test).

These results were compared to clustering with the same methodology (k-means clustering with K = 3, knee method) using inferred immune cell content instead of raw expression. K-means assigned patients to three subgroups: sub0, sub1, and sub2. As can be seen in Table 2, sub0 was highly and significantly enriched with EGFR-mutated patients: 33 patients in sub0 (20.1%) were EGFR-mutated, compared to 5.3% in sub1 and 9.5% in sub2 (*p* = 6 × 10^−4^, chi-square test).

**Figure 3:**
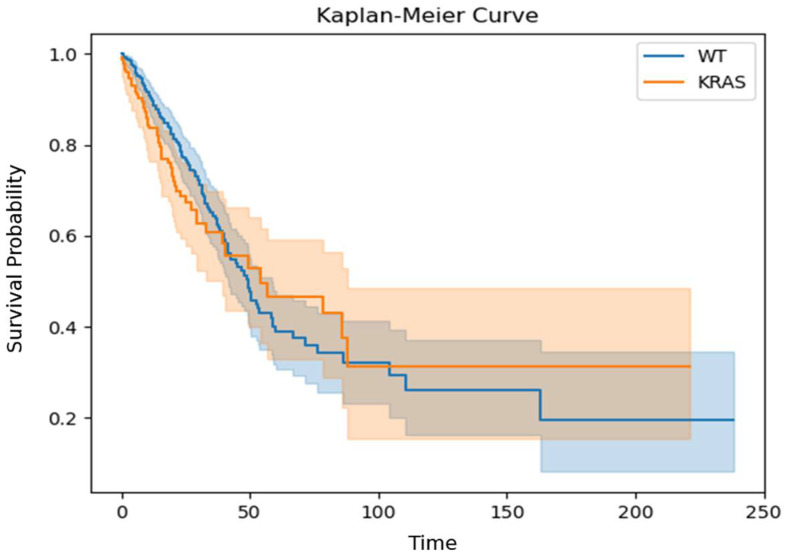
Kaplan–Meier curve of KRAS-mutated and KRAS-WT patients. The Y-axis shows the survival probability, while the X-axis represents the respective time interval in months. Separate lines, in different colors, each with a band of the same color, represent the comparison of the KRAS-WT (n = 347) and KRAS-mutated patients (n = 151); *p* = 0.45; log-rank test.

**Figure 4:**
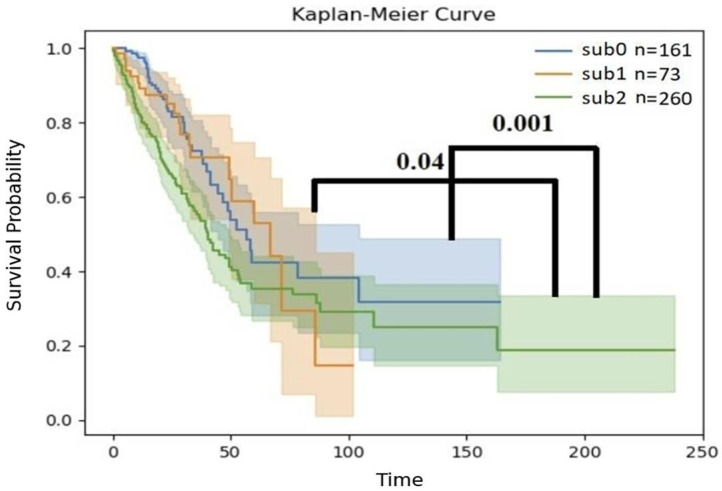
Kaplan–Meier curve of the sub0, sub1, and sub2 groups of patients. Statistical comparisons, performed with the log-rank method [30], are shown as black lines. Four individuals were omitted due to a lack of RNA expression profiles.

To assess whether these groups were clinically different, the survival of patients from the three groups was compared using a Kaplan–Meier plot (Figure 3). The groups showed distinct survival patterns: patients from sub2, which was enriched with EGFR-WT (n = 260), had a significantly worse prognosis than those in the sub0 group (*p*-value = 0.001, log-rank test) or sub1 group (*p*-value = 0.04, log-rank test) (Figure 4).

### 3.3. Differential Expression of Immune Checkpoints Between Immune Cell Clusters

**Figure 5:**
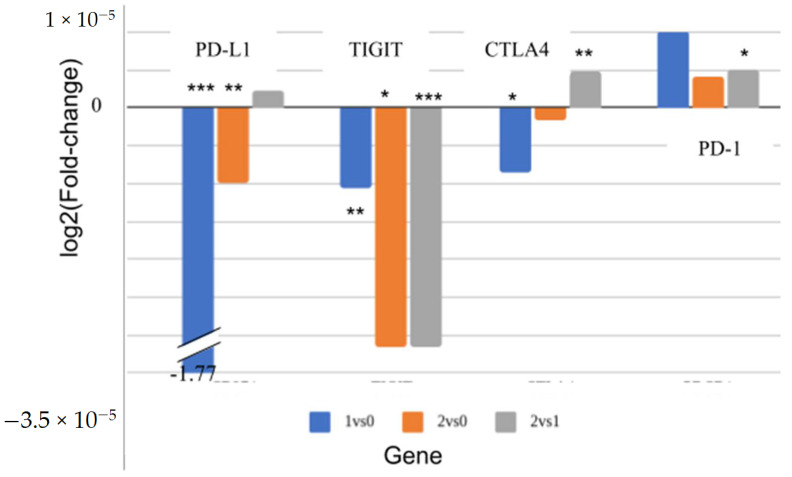
Differences in immune checkpoint expression between the immunity-defined subgroups. The values are minus log normalized with base 2 of the mean fold change. Asterisks indicate the significance of the difference (*** *p* < 0.001; ** *p* < 0.01; * *p* < 0.05, *t*-test).

We then compared the expression of known immune checkpoints between the different groups (Figure 5). Since these groups were defined by their abundance of immune cells, it was possible they would also differ in immune checkpoint expression. Since these were the targets of immune checkpoint inhibitors, we were interested in comparing their expression between the subgroups. Significant differences were observed for four targets: T cell immunoreceptor with Ig and ITIM domains (TIGIT) expression differed in all three comparisons: between sub1 and sub0 (*p* = 0.01), between sub2 and sub0 (*p* = 0.01), and between sub2 and sub1 (*p* = 7 × 10^−4^). Programmed death-ligand 1 (PD-L1; CD274) expression differed in two comparisons, sub1/sub0 (*p* = 2.54 × 10^−7^) and sub2/sub0 (*p* = 0.004), as did Cytotoxic T-lymphocyte associated protein 4 (CTLA4; *p* = 0.02 and *p* = 0.003, respectively). Programmed cell death protein 1 (PD-1; PDCD-1) expression differed only between sub2 and sub1 (*p* = 0.03). No other known immune checkpoints were significantly different between any of the clusters.

Finally, we tested the hypothesis that the four EGFR-mutated patients were wrongly assigned to sub1. If this were the case, we would expect little difference between the EGFR-mutated patients assigned to sub1 (n = 4) and the EGFR-mutated patients in the other clusters. A differential expression (DE) comparison between the EGFR-mutated patients in each subgroup revealed significant results (*p* < 0.05, FDR < 0.25) in both comparisons, 1428 genes significantly differed between the EGFR-mutated patients from sub0 and EGFR-mutated patients from sub1, and 510 genes differed between sub2 and sub1 patients. For comparison, four patients were randomly chosen from sub0 or sub2 and compared using the same procedure to the remainder of the EGFR-mutated patients in the same cluster (see Methods). This process was repeated 18 times to give the average and standard deviation of the differences expected if these four patients in sub1 had mistakenly been assigned to it. Fewer genes were significantly different within either of the subgroups (77 ± 149.2 and 117 ± 266.8 results were obtained for sub0 and sub2, respectively), suggesting that EGFR-mutated patients were randomly assigned to sub1, although the difference was significant for sub0 (*p* < 1.23 × 10^−18^, z-test) but not for sub2 patients (*p* = 0.15, z-test).

## 4. Discussion

In this work, we used inferred immune content rather than expression patterns to divide LUAD patients. This approach revealed clusters that were more strongly associated with mutation type (Table 2), that were distinct in terms of survival (Figure 4), and that had differentially expressed immune checkpoint genes (Figure 5).

Inferring cell profiles using xCell proved to be informative in lung cancer (as discussed below). It is important to test the same in other cancers: immune cell invasion was shown to correlate with progression in lung cancer and in many other cancer types [36,37]. The approach presented here could offer a simple way to investigate immune invasion in many cancers. It suggests that RNA-seq, rather than many specific staining approaches, could be used to understand how the infiltration of different cells helps or attacks cancer cells.

The immune uniqueness of tumors harboring EGFR or KRAS mutations in terms of immune response is poorly characterized, but the differences in the demographics of the two populations have been reported [13,14]. When it comes to treatment, LUAD patients are divided by their driver mutations, although the therapeutic usefulness of the traditional division in the face of immunotherapy has recently been challenged [31]. The use of immunotherapy has dramatically increased the need for a new and more precise way to divide LUAD patients. To the best of our knowledge, this is the first time an unsupervised machine learning method has been applied to divide LUAD patients in TCGA based on their inferred immune profiles.

Since the clustering approach presented here is based on (inferred) immune profiles, it is not surprising to see large differences in the expression of immune checkpoints. Accordingly, PDCD-1, which is expressed in activated T cells, was overexpressed in sub1 compared to sub0. This suggests that the tumors in this cluster had a higher number of activated T cells. In addition, inhibiting the PD-1/PD-L1 axis, the main target of most of the immune checkpoint inhibitors that were used, was expected to be more helpful to patients in the sub1 cluster. This is in agreement with our finding that sub1 tumors rarely carry EGFR mutations. The immune checkpoint inhibitors were shown to be less valuable for patients with EGFR mutations [31]. In other words, tumors with EGFR mutations did not attract the immune response that EGFR-WT tumors attracted, and as a result, they were both less susceptible to immune checkpoint inhibitors (ICIs) and tended to fall into unique immune profile clusters. EGFR-mutated tumors were grouped mostly in sub0, and they constituted more than 20% of that group (Table 2). The finding that some of the EGFR-WT tumors clustered together with EGFR-mutated cases suggests that they attract the same immune response as EGFR-mutated tumors. This is in accordance with the fact that most EGFR-WT patients do not respond to ICIs [32]. The EGFR-WT tumors that do not respond to ICIs may be more similar in their immune profiles to EGFR-mutated patients, which are often too restrictive to ICI treatment. The division we present here may be the first step toward finding a division that can guide ICI therapy based on an immune profiling approach.

Our study population, taken from TCGA, is consistent in some respects with previous reports. For example, EGFR mutations tend to occur more frequently in patients who have never smoked than in smokers [38]. Unexpected differences were found, as previously reported, in the overall survival of both the KRAS- and EGFR-mutated groups versus the WT group (Figure 1 and Figure 2). These differences are proposed to result from a biased TCGA LUAD population in which most of the patients had their disease identified at an early stage.

However, in other aspects, the TCGA population was abnormal. The overwhelming majority of patients included in this study were diagnosed in Stage 1, while most lung adenocarcinomas are usually diagnosed at later stages [39].

This work is based on unsupervised machine learning (i.e., clustering) [40,41]. Unlike previous works, we used inferred immune cell estimates to cluster tumors together. We used xCell to project RNA-seq-based expression profiles into inferred immune profiles. While numerous other methods have been proposed to infer immune cell content from bulk RNA-seq [42,43,44], a complete description of all these methods was beyond the scope of this work. However, repeating the analysis with other immune inference methods gave similar results (see Table S1), suggesting that our findings do not depend on the choice of the immune inference method.

We bring several lines of evidence that support the conclusion that classifying patients by inferred immune profiles divides them into biologically meaningful groups: (i) When patients were clustered based on expression without immune inference, a weak non-random bias in KRAS-positive patients to one particular cluster was observed (Table S2), while EGFR-positive patients were randomly distributed among the clusters. On the other hand, when inferred immune cell counts were used for clustering, EGFR-mutated patients, but not KRAS-mutated patients, were distributed non-randomly between the clusters with a more distinct bias (Table 2: sub2 vs. sub0, *p* = 0.002; sub1 vs. sub0, *p* = 0.003). (ii) The clusters formed with immune inference differed in overall survival (Figure 4), with sub2 differing significantly from sub1 and sub0. (iii) Highly significant differences were observed in the expression levels of some of the targets of immune checkpoint inhibitors (e.g., PD-L1, which differed between sub1 and sub0) (Figure 5). It should be noted that both the EGFR and KRAS mutation information was taken from TCGA LUAD data.

Based on previous reports [45], enhanced PD-L1 expression on tumor cells leads to T-cell exhaustion, promoting tumor proliferation and survival [46]. Thus, an immune checkpoint inhibitor (e.g., anti-PD-L1) may assist in releasing the T cells from exhaustion. High PD-L1 expression has been approved by the Food and Drug Administration (FDA) as a biomarker [47]. We propose that the patients from this sub0 cluster are less likely to respond to ICIs that target the PD-1/PD-L1 axis since they utilize other immune checkpoints for immune evasion (e.g., TIGIT), but the other clusters may better respond to PD-1/PD-L1 inhibitors.

The main significance of this study is in showing immune cell content can be inferred from bulk RNA-seq with sufficient accuracy to promote subtype detection and perhaps a better understanding of cancer. We only applied this approach to lung adenocarcinoma, but in the future, it could be interesting to test other cancer types and other inference methods. One of the limitations of this study is that it relies on the immune inference of xCell for classification. Other immune inference methods were not rigorously pursued.

Methods for directly measuring immune profiles have been suggested [48,49]. It would be interesting to examine clustering on actual, rather than inferred, immune profiles to cluster LUAD patients.

## 5. Conclusions

To conclude, we show that immune inference can uncover new clusters of LUAD patients that are otherwise hidden. We directly show that using immune inference gives more compact subgroups and that this division is clinically meaningful. We also show that these clusters differ molecularly, at the very least, in their expression of ICI targets. Specifically, we show that PD-L1 is markedly lower in one cluster while TIGIT is lower in another.

## Figures and Tables

**Table 1:** Demographic characteristics of TCGA LUAD populations.

	Characteristics	EGFR Status		*p*-Val WT-Mut
		WT	Mut	
Tumor grade	Population (n)	492	66	
	Grade I	247	29	0.65
	Grade II	105	14	0.89
	Grade III	70	13	0.42
	Grade IV	21	6	0.19
	Not available	49	4	
Smoking	Yes	386	34	0.07
	No	46	27	$3.26\times 10^{-8}$
	Not available	60	5	
Diagnosis age (years)	≤60	140	17	0.84
	>60	289	42	0.78
	Not available	63	7	
Gender	Female	230	43	0.14
	Male	215	19	0.16
	Not available	47	4	
Race	White	336	46	0.99
	Black	46	6	0.87
	Asian	5	3	0.1
	Other	1	0	1
	Not available	104	11	

**Table 2:** The distribution of patients (by EGFR and KRAS status) in different patient groups. The distribution of mutations (EGFR and KRAS status) in different patient clusters. EGFR and KRAS mutations were counted after k-means clustering into “sub” groups. In some cells, the fraction of patients carrying a mutation is shown in parentheses. Four individuals were omitted due to a lack of RNA expression profiles.

	Sub0	Sub1	Sub2
EGFR-WT	131	71	239
EGFR-mutated	33 (20.1%)	4 (5.3%)	25 (9.5%)
KRAS-WT	178	59	116
KRAS-mutated	86 (32.6%)	16 (21.3%)	48 (29%)

## Data Availability

All the data are available upon reasonable request.

## Supplementary Materials

The following supporting information can be downloaded at https://www.mdpi.com/article/10.3390/biomedicines13040849/s1, Table S1: PCA of other methods; Table S2: Comparison of clustering with and without immune inference.

## References

[1] Ferlay J., Colombet M., Soerjomataram I., Parkin D.M., Piñeros M., Znaor A., Bray F. (2021). Cancer Statistics for the Year 2020: An Overview. Int. J. Cancer.

[2] Zappa C., Mousa S.A. (2016). Non-Small Cell Lung Cancer: Current Treatment and Future Advances. Transl. Lung Cancer Res..

[3] Meng F., Zhang L., Ren Y., Ma Q. (2019). The Genomic Alterations of Lung Adenocarcinoma and Lung Squamous Cell Carcinoma Can Explain the Differences of Their Overall Survival Rates. J. Cell Physiol..

[4] Unni A.M., Lockwood W.W., Zejnullahu K., Lee-Lin S.-Q., Varmus H. (2015). Evidence That Synthetic Lethality Underlies the Mutual Exclusivity of Oncogenic KRAS and EGFR Mutations in Lung Adenocarcinoma. eLife.

[5] Shen M., Qi R., Ren J., Lv D., Yang H. (2022). Characterization with KRAS Mutant Is a Critical Determinant in Immunotherapy and Other Multiple Therapies for Non-Small Cell Lung Cancer. Front. Oncol..

[6] Ferrer I., Zugazagoitia J., Herbertz S., John W., Paz-Ares L., Schmid-Bindert G. (2018). KRAS-Mutant Non-Small Cell Lung Cancer: From Biology to Therapy. Lung Cancer.

[7] Gu M., Xu T., Chang P. (2021). KRAS/LKB1 and KRAS/TP53 Co-Mutations Create Divergent Immune Signatures in Lung Adenocarcinomas. Ther. Adv. Med. Oncol..

[8] Kim J.H., Kim H.S., Kim B.J. (2017). Oncotarget Prognostic Value of KRAS Mutation in Advanced Non-Small-Cell Lung Cancer Treated with Immune Checkpoint Inhibitors: A Meta-Analysis and Review. Oncotarget.

[9] Wee P., Wang Z. (2017). Epidermal Growth Factor Receptor Cell Proliferation Signaling Pathways. Cancers.

[10] Russo A., Franchina T., Ricciardi G., Battaglia A., Picciotto M., Adamo V. (2019). Heterogeneous Responses to Epidermal Growth Factor Receptor (EGFR) Tyrosine Kinase Inhibitors (TKIs) in Patients with Uncommon EGFR Mutations: New Insights and Future Perspectives in This Complex Clinical Scenario. Int. J. Mol. Sci..

[11] Baek J.H., Sun J.M., Min Y.J., Cho E.K., Cho B.C., Kim J.H., Ahn M.J., Park K. (2015). Efficacy of EGFR Tyrosine Kinase Inhibitors in Patients with EGFR-Mutated Non-Small Cell Lung Cancer except Both Exon 19 Deletion and Exon 21 L858R: A Retrospective Analysis in Korea. Lung Cancer.

[12] Lin A., Wei T., Meng H., Luo P., Zhang J. (2019). Role of the Dynamic Tumor Microenvironment in Controversies Regarding Immune Checkpoint Inhibitors for the Treatment of Non-Small Cell Lung Cancer (NSCLC) with EGFR Mutations. Mol. Cancer.

[13] Langer C.J. (2011). Roles of EGFR and KRAS Mutations in the Treatment of Patients with Non–Small-Cell Lung Cancer. Pharm. Ther..

[14] Cucurull M., Notario L., Sanchez-Cespedes M., Hierro C., Estival A., Carcereny E., Saigí M. (2022). Targeting KRAS in Lung Cancer Beyond KRAS G12C Inhibitors: The Immune Regulatory Role of KRAS and Novel Therapeutic Strategies. Front. Oncol..

[15] Takamochi K., Oh S., Suzuki K. (2013). Differences in EGFR and KRAS Mutation Spectra in Lung Adenocarcinoma of Never and Heavy Smokers. Oncol. Lett..

[16] Aran D., Hu Z., Butte A.J. (2017). XCell: Digitally Portraying the Tissue Cellular Heterogeneity Landscape. Genome Biol..

[17] Chen Y., Chen H., Mao B., Zhou Y., Shi X., Tang L., Jiang H., Wang G., Zhuang W. (2019). Transcriptional Characterization of the Tumor Immune Microenvironment and Its Prognostic Value for Locally Advanced Lung Adenocarcinoma in a Chinese Population. Cancer Manag. Res..

[18] Cerami E., Gao J., Dogrusoz U., Gross B.E., Sumer S.O., Aksoy B.A., Jacobsen A., Byrne C.J., Heuer M.L., Larsson E. (2012). The CBio Cancer Genomics Portal: An Open Platform for Exploring Multidimensional Cancer Genomics Data. Cancer Discov..

[19] Gao J., Aksoy B.A., Dogrusoz U., Dresdner G., Gross B., Sumer S.O., Sun Y., Jacobsen A., Sinha R., Larsson E. (2013). Integrative Analysis of Complex Cancer Genomics and Clinical Profiles Using the CBioPortal. Sci. Signal.

[20] Ghosh D., Vogt A. (2012). Outliers: An Evaluation of Methodologies. Jt. Stat. Meet..

[21] MuTect2 Variant Aggregation and Masking. https://xenabrowser.net/datapages/?dataset=TCGA-LUAD.mutect2_snv.tsv%20&host=https%3A%2F%2Fgdc.xenahubs.net&removeHub=https%3A%2F%2Fxena.treehouse.gi.ucsc.edu%3A443.

[22] Satopää V., Albrecht J., Irwin D., Raghavan B. Finding a “Kneedle” in a Haystack: Detecting Knee Points in System Behavior. Proceedings of the 2011 31st International Conference on Distributed Computing Systems Workshops.

[23] Goldman M.J., Craft B., Hastie M., Repečka K., McDade F., Kamath A., Banerjee A., Luo Y., Rogers D., Brooks A.N. (2020). Visualizing and Interpreting Cancer Genomics Data via the Xena Platform. Nat. Biotechnol..

[24] R Core Team (2021). R: A Language and Environment for Statistical Computing.

[25] Love M.I., Huber W., Anders S. (2014). Moderated Estimation of Fold Change and Dispersion for RNA-Seq Data with DESeq2. Genome Biol..

[26] Li B., Severson E., Pignon J.C., Zhao H., Li T., Novak J., Jiang P., Shen H., Aster J.C., Rodig S. (2016). Comprehensive Analyses of Tumor Immunity: Implications for Cancer Immunotherapy. Genome Biol..

[27] Weinstein J.N., Collisson E.A., Mills G.B., Shaw K.R.M., Ozenberger B.A., Ellrott K., Sander C., Stuart J.M., Chang K., Creighton C.J. (2013). The Cancer Genome Atlas Pan-Cancer Analysis Project. Nat. Genet..

[28] Graham R.P., Treece A.L., Lindeman N.I., Vasalos P., Shan M., Jennings L.J., Rimm D.L. (2018). Worldwide Frequency of Commonly Detected EGFR Mutations. Arch. Pathol. Lab. Med..

[29] Likas A., Vlassis N., Verbeek J.J. (2003). The Global K-Means Clustering Algorithm. Pattern Recognit..

[30] Koletsi D., Pandis N. (2017). Survival Analysis, Part 2: Kaplan-Meier Method and the Log-Rank Test. Am. J. Orthod. Dentofac. Orthop..

[31] To K.K.W., Fong W., Cho W.C.S. (2021). Immunotherapy in Treating EGFR-Mutant Lung Cancer: Current Challenges and New Strategies. Front. Oncol..

[32] Hastings K., Yu H.A., Wei W., Sanchez-Vega F., Deveaux M., Choi J., Rizvi H., Lisberg A., Truini A., Lydon C.A. (2019). EGFR Mutation Subtypes and Response to Immune Checkpoint Blockade Treatment in Non-Small-Cell Lung Cancer. Ann. Oncol..

[33] Pedregosa F., Varoquaux G., Gramfort A., Michel V., Thirion B., Grisel O., Blondel M., Prettenhofer P., Weiss R., Dubourg V. (2011). Scikit-learn: Machine Learning in Python. J. Mach. Learn. Res..

[34] Deng M., Brägelmann J., Kryukov I., Saraiva-Agostinho N., Perner S. (2017). FirebrowseR: An R client to the Broad Institute’s Firehose Pipeline. Database.

[35] Hunter J.D. (2007). Matplotlib: A 2D graphics environment. Comput. Sci. Eng..

[36] Gentles A.J., Newman A.M., Liu C.L., Bratman S.V., Feng W., Kim D., Nair V.S., Xu Y., Khuong A., Hoang C.D. (2015). The prognostic landscape of genes and infiltrating immune cells across human cancers. Nat. Med..

[37] Bagaev A., Kotlov N., Nomie K., Svekolkin V., Gafurov A., Isaeva O., Osokin N., Kozlov I., Frenkel F., Gancharova O. (2021). Conserved pan-cancer microenvironment subtypes predict response to immunotherapy. Cancer Cell.

[38] Luo W., Tian P., Wang Y., Xu H., Chen L., Tang C., Shu Y., Zhang S., Wang Z., Zhang J. (2018). Characteristics of Genomic Alterations of Lung Adenocarcinoma in Young Never-Smokers. Int. J. Cancer.

[39] Chen Y., Jin L., Jiang Z., Liu S., Feng W. (2021). Identifying and Validating Potential Biomarkers of Early Stage Lung Adenocarcinoma Diagnosis and Prognosis. Front. Oncol..

[40] Toedt G., Barbus S., Wolter M., Felsberg J., Tews B., Blond F., Sabel M.C., Hofmann S., Becker N., Hartmann C. (2011). Molecular Signatures Classify Astrocytic Gliomas by IDH1 Mutation Status. Int. J. Cancer.

[41] Plath M., Gass J., Hlevnjak M., Li Q., Feng B., Hostench X.P., Bieg M., Schroeder L., Holzinger D., Zapatka M. (2021). Unraveling Most Abundant Mutational Signatures in Head and Neck Cancer. Int. J. Cancer.

[42] Liu X., Chen W., Fang Y., Yang S., Chang L., Chen X., Ye H., Tang X., Zhong S., Zhang W. (2021). ADEIP: An Integrated Platform of Age-Dependent Expression and Immune Profiles across Human Tissues. Brief. Bioinform..

[43] Alldredge J., Randall L., de Robles G., Agrawal A., Mercola D., Liu M., Randhawa P., Edwards R., McClelland M., Rahmatpanah F. (2020). Transcriptome Analysis of Ovarian and Uterine Clear Cell Malignancies. Front. Oncol..

[44] Li J., Chen H., Guo H., Qiu M., Yang F. (2020). Characterization of Gene Expression Profiles of Esophageal Cancer Patients with Different Nonsynonymous Tumor Mutation Burden. Thorac. Cancer.

[45] Zhang J., Han X., Lin L., Chen J., Wang F., Ding Q., Hao L., Wang L., Wei J., Wang Y. (2022). Unraveling the Expression Patterns of Immune Checkpoints Identifies New Subtypes and Emerging Therapeutic Indicators in Lung Adenocarcinoma. Oxid. Med. Cell Longev..

[46] Wang Q., Li M., Yang M., Yang Y., Song F., Zhang W., Li X., Chen K. (2020). Analysis of Immune-Related Signatures of Lung Adenocarcinoma Identified Two Distinct Subtypes: Implications for Immune Checkpoint Blockade Therapy. Aging.

[47] Doroshow D.B., Bhalla S., Beasley M.B., Sholl L.M., Kerr K.M., Gnjatic S., Wistuba I.I., Rimm D.L., Tsao M.S., Hirsch F.R. (2021). PD-L1 as a Biomarker of Response to Immune-Checkpoint Inhibitors. Nat. Rev. Clin. Oncol..

[48] Gustafson M.P., Lin Y., LaPlant B., Liwski C.J., Maas M.L., League S.C., Bauer P.R., Abraham R.S., Tollefson M.K., Kwon E.D. (2013). Immune Monitoring Using the Predictive Power of Immune Profiles. J. Immunother. Cancer.

[49] Michaud D.S., Houseman E.A., Marsit C.J., Nelson H.H., Wiencke J.K., Kelsey K.T. (2015). Understanding the Role of the Immune System in the Development of Cancer: New Opportunities for Population-Based Research. Cancer Epidemiol. Biomark. Prev..

