# Supplementary material for: Immune Clustering Reveals Molecularly Distinct Subtypes of Lung Adenocarcinoma
Source: Biomedicines. 2025 Apr 2;13(4):849. doi: 10.3390/biomedicines13040849 (PMC12024753; doi:10.3390/biomedicines13040849)
Supplement: Supplementary file 1 [file biomedicines-13-00849-s001.zip › biomedicines-3509226-supplementary.pdf]

biomedicines

MDPI

Supplementary Materials

Table S1. PCA of other methods. Because each method uses its own way to estimate the immune profile, the score may be arbitrary. Positive scores indicate an immune impact. The higher the score, the higher the impact and vice versa.

Cell type	EPIC	TIMER	CIBERSORT	CIBERSORT-ABS	QUANTISEQ	MCPCOUNTER	XCELL
**PC1**							
**Cancer associated fibroblast**	0.59	ND	ND	ND	ND	0.14	ND
**T cell CD8+**	-0.49	0.31	-0.25	0.36	-0.23	0.36	0.24
**T cell CD4+**	-0.34	0.47	ND	ND	ND	ND	ND
**NK cell**	0.05	ND	ND	ND	-0.16	ND	ND
**Endothelial cell**	0.05	ND	ND	ND	ND	0.12	ND
**B cell**	0	0.48	ND	ND	-0.24	0.27	0.25
**Macrophage**	0	0.14	ND	ND	ND	ND	ND
**Neutrophil**	ND	0.34	ND	ND	-0.17	-0.11	ND
**Mast cell activated**	ND	ND	0.42	ND	ND	ND	ND
**Myeloid dendritic cell resting**	ND	ND	0.35	ND	ND	ND	ND
**Monocyte**	ND	ND	0.34	ND	0.09	0.47	ND
**Macrophage M2**	ND	ND	0.3	0.31	-0.31	ND	ND
**NK cell resting**	ND	ND	-0.27	0.31	ND	ND	ND
**Macrophage M1**	ND	ND	-0.24	0.39	-0.4	ND	ND
**Macrophage M0**	ND	ND	-0.22	ND	ND	ND	ND
**T cell follicular helper**	ND	ND	-0.22	ND	ND	ND	ND
**T cell CD4+ memory activated**	ND	ND	-0.21	0.23	ND	ND	ND
**T cell CD4+ memory resting**	ND	ND	ND	0.34	ND	ND	ND
**T cell regulatory (Tregs)**	ND	ND	ND	0.28	-0.46	ND	ND
**B cell memory**	ND	ND	ND	0.24	ND	ND	0.22
**B cell naive**	ND	ND	ND	0.21	ND	ND	ND
**Myeloid dendritic cell**	ND	ND	ND	ND	0.23	0.25	0.24
**Macrophage/Monocyte**	ND	ND	ND	ND	ND	0.47	ND
**T cell CD8+ central memory**	ND	ND	ND	ND	ND	ND	0.26
**T cell CD4+ naive**	ND	ND	ND	ND	ND	ND	0.23
**Plasmacytoid dendritic cell**	ND	ND	ND	ND	ND	ND	0.23
**Class-switched memory B cell**	ND	ND	ND	ND	ND	ND	0.21
**PC2**							
**Endothelial cell**	0.53	ND	ND	ND	ND	-0.31	ND
**B cell**	0.46	-0.38	ND	ND	-0.52	0.28	ND
**Macrophage**	0.44	0.58	ND	ND	ND	ND	ND
**T cell CD4+**	0.41	-0.44	ND	ND	ND	ND	ND
**T cell CD8+**	0.16	0.48	-0.27	ND	-0.3	0.44	ND
**NK cell**	-0.06	ND	ND	ND	-0.06	0.42	ND
**Cancer associated fibroblast**	0.03	ND	ND	ND	ND	-0.17	0.26
**Myeloid dendritic cell**	0.57	0.11	-0.17	0.28	ND	-0.31	ND
**Neutrophil**	ND	0.29	0.27	ND	0.51	-0.3	ND
**NK cell activated**	ND	ND	-0.44	0.29	ND	ND	ND
**T cell follicular helper**	ND	ND	-0.39	ND	ND	ND	ND
**Macrophage M2**	ND	ND	0.31	0.24	-0.07	ND	0.19
**NK cell resting**	ND	ND	0.3	-0.15	ND	ND	ND
**T cell CD4+ memory resting**	ND	ND	0.26	0.15	ND	ND	ND
**Mast cell activated**	ND	ND	-0.25	0.49	ND	ND	ND
**Mast cell resting**	ND	ND	0.23	ND	ND	ND	ND
**Myeloid dendritic cell resting**	ND	ND	ND	0.44	ND	ND	ND
**Monocyte**	ND	ND	ND	0.41	0.31	-0.29	ND
**Macrophage M0**	ND	ND	ND	-0.24	ND	ND	ND
**B cell plasma**	ND	ND	ND	-0.16	ND	ND	ND
**Macrophage M1**	ND	ND	ND	ND	0.34	ND	ND
**T cell CD4+ (non-regulatory)**	ND	ND	ND	ND	-0.28	ND	ND
**uncharacterized cell**	ND	ND	ND	ND	-0.21	ND	ND
**T cell regulatory (Tregs)**	ND	ND	ND	ND	-0.2	ND	ND
**Macrophage/Monocyte**	ND	ND	ND	ND	ND	-0.29	ND
**T cell**	ND	ND	ND	ND	ND	0.27	ND
**Hematopoietic stem cell**	ND	ND	ND	ND	ND	ND	0.32
**T cell CD4+ Th2**	ND	ND	ND	ND	ND	ND	-0.31
**Endothelial cell**	ND	ND	ND	ND	ND	ND	0.24
**T cell CD8+ naive**	ND	ND	ND	ND	ND	ND	-0.22
**T cell CD4+ Th1**	ND	ND	ND	ND	ND	ND	-0.22
**Common lymphoid progenitor**	ND	ND	ND	ND	ND	ND	-0.2
**Plasmacytoid dendritic cell**	ND	ND	ND	ND	ND	ND	-0.19

 https://doi.org/10.3390/biomedicines13040849

Table S2. Comparison of clustering with and without immune inference. Left side: patients are clustered based on expression without immune inference. Right side: patients are clustered after immune inference. The number of clusters is dependent on the uniqueness of each method of estimation of immune inference. Four individuals were omitted due to a lack of RNA expression profiles.

	Immune Inference				
	Without			With	
KRAS					
Sub0	Mutated	63	Sub0	Mutated	48
	WT	146		WT	116
Sub1	Mutated	8	Sub1	Mutated	16
	WT	40		WT	59
Sub2	Mutated	57	Sub2	Mutated	86
	WT	94		WT	178
Sub3	Mutated	22			
	WT	73			
EGFR					
Sub0	Mutated	30	Sub0	Mutated	33
	WT	179		WT	131
Sub1	Mutated	6	Sub1	Mutated	4
	WT	89		WT	71
Sub2	Mutated	18	Sub2	Mutated	25
	WT	133		WT	239
Sub3	Mutated	8			
	WT	40			
